# Association of Regional White Matter Hyperintensities With Longitudinal Alzheimer-Like Pattern of Neurodegeneration in Older Adults

**DOI:** 10.1001/jamanetworkopen.2021.25166

**Published:** 2021-10-05

**Authors:** Batool Rizvi, Patrick J. Lao, Anthony G. Chesebro, Jordan D. Dworkin, Erica Amarante, Juliet M. Beato, Jose Gutierrez, Laura B. Zahodne, Nicole Schupf, Jennifer J. Manly, Richard Mayeux, Adam M. Brickman

**Affiliations:** 1 Taub Institute for Research on Alzheimer’s Disease and the Aging Brain, Vagelos College of Physicians and Surgeons, Columbia University, New York, New York; 2 Department of Psychiatry, Vagelos College of Physicians and Surgeons, Columbia University, New York, New York; 3 Department of Biostatistics, Mailman School of Public Health, Columbia University, New York, New York; 4 Department of Psychology, University of Michigan, Ann Arbor; 5 Gertrude H. Sergievsky Center, Vagelos College of Physicians and Surgeons, Columbia University, New York, New York; 6 Department of Neurology, Vagelos College of Physicians and Surgeons, Columbia University, New York, New York; 7 Department of Epidemiology, Mailman School of Public Health, Columbia University, New York, New York

## Abstract

**Importance:**

Small vessel cerebrovascular disease, visualized as white matter hyperintensities (WMH), is associated with cognitive decline and risk of clinical Alzheimer disease (AD). One way in which small vessel cerebrovascular disease could contribute to AD is through the promotion of neurodegeneration; the effect of small vessel cerebrovascular disease on neurodegeneration may differ across racial and ethnic groups.

**Objective:**

To examine whether WMH volume is associated with cortical thinning over time and subsequent memory functioning and whether the association between WMH volume and cortical thinning differs among racial and ethnic groups.

**Design, Setting, and Participants:**

This longitudinal community-based cohort study included older adults from northern Manhattan who were participants in the Washington Heights–Inwood Columbia Aging Project. Participants underwent two 3T magnetic resonance imaging (MRI) scans a mean of 4 years apart. Data were collected from March 2011 to January 2020.

**Exposures:**

Total and regional WMH volumes.

**Main Outcomes and Measures:**

The association of total and regional WMH volumes with cortical thinning over time was tested using general linear models in a vertexwise analysis. Cortical thinning was measured vertexwise by symmetrized percent change between 2 time points. The association of changes in cortical thickness with memory and whether this association differed by race and ethnicity was also analyzed. Delayed memory was a secondary outcome.

**Results:**

In 303 participants (mean [SD] age, 73.16 [5.19] years, 181 [60%] women, 96 [32%] non-Hispanic White, 113 [37%] Non-Hispanic Black, 94 [31%] Hispanic), baseline WMH volumes were associated with cortical thinning in medial temporal and frontal/parietal regions. Specifically, total WMH volume was associated with cortical thinning in the right caudal middle frontal cortex (*P* = .001) and paracentral cortex (*P* = .04), whereas parietal WMH volume was associated with atrophy in the left entorhinal cortex (*P* = .03) and right rostral middle frontal (*P* < .001), paracentral (*P* < .001), and pars triangularis (*P* = .02) cortices. Thinning of the right caudal middle frontal and left entorhinal cortices was related to lower scores on a memory test administered closest to the second MRI visit (right caudal middle frontal cortex: standardized β = 0.129; unstandardized *b* = 0.335; 95% CI, 0.055 to 0.616; *P* = .01; left entorhinal cortex: β = 0.119; *b* = 0.290; 95% CI, 0.018 to 0.563; *P* = .03). The association of total WMH with thinning in the right caudal middle frontal and right paracentral cortex was greater in non-Hispanic Black participants compared with White participants (right caudal middle frontal cortex: β = −0.222; *b* = −0.059; 95% CI, −0.114 to −0.004; *P* = .03; right paracentral cortex: β = −0.346; *b* = −0.155; 95% CI, −0.244 to −0.066; *P* = .001). The association of parietal WMH with cortical thinning of the right rostral middle frontal, right pars triangularis, and right paracentral cortices was also stronger among non-Hispanic Black participants compared with White participants (right rostral middle frontal cortex: β = −0.252; *b* = −0.202; 95% CI, −0.349 to −0.055; *P* = .007; right pars triangularis cortex: β = −0.327; *b* = −0.253; 95% CI, −0.393 to −0.113; *P* < .001; right paracentral cortex: β = −0.263; *b* = −0.337; 95% CI, −0.567 to −0.107; *P* = .004).

**Conclusions and Relevance:**

In this study, small vessel cerebrovascular disease, operationalized as WMH, was associated with subsequent cortical atrophy in regions that overlap with typical AD neurodegeneration patterns, particularly among non-Hispanic Black older adults. Cerebrovascular disease may affect risk and progression of AD by promoting neurodegeneration and subsequent memory decline.

## Key Points

**Question:**

What is the association of small vessel cerebrovascular disease, operationalized as white matter hyperintensities (WMH), with cortical thinning in a racially and ethnically diverse population of older adults?

**Findings:**

In this cohort study of 303 older adults, increased parietal WMH volume was associated with left entorhinal cortex thinning over 4 years, while both total and parietal WMH were associated with frontal and parietal cortical thinning; cortical thinning, in turn, was associated with worse memory. The association between WMH and cortical thinning was strongest among Black participants.

**Meaning:**

In this study, WMH were associated with cortical thinning in older adults, particularly non-Hispanic Black participants, in a pattern similar to the neurodegeneration in early stages of Alzheimer disease.

## Teaser

This cohort study examines whether white matter hyperintensity volume is associated with cortical thinning and subsequent memory functioning and whether the association between white matter hyperintensity volume and cortical thinning differs among racial and ethnic groups.

## Introduction

Small vessel cerebrovascular disease manifests radiologically as white matter hyperintensities (WMH), areas of increased brightness on T2-weighted magnetic resonance imaging (MRI) scans. White matter hyperintensities volume is strongly associated with poorer cognition in normal aging,^1,2,3,4^ but there is also emerging evidence that it plays a role in the clinical expression of Alzheimer disease (AD). Individuals diagnosed with mild cognitive impairment (MCI), those diagnosed with frank clinical AD, and those at genetic risk of development of AD have elevated WMH volume compared with control participants.^5,6,7,8,9,10^ Increased WMH volume, particularly when distributed in the parietal lobes, predicts future development of clinical AD and a cognitive impairment profile that is phenotypically similar to what is seen in AD, including memory and executive dysfunction.^6,10,11,12^

One possible way in which WMH—or the underlying ischemic disease they represent—may affect the onset and course of AD is through their impact on cortical neurodegeneration. There is cross-sectional evidence that WMH volume correlates with atrophy patterns that overlap spatially with those observed in typical normal aging and in AD.^13,14,15,16,17^ Our previous work showed that the association between WMH severity and memory is statistically mediated in part by regional cortical thickness, suggesting that WMH could lead to an AD-like amnestic syndrome through their effect on distributed cortical atrophy.^15^ Although there are reports that vascular risk factors, such as hypertension, promote increased rates of cortical thinning,^18,19,20^ there are few studies that examined the association of WMH with cortical atrophy over time, and the findings have been mixed.^21,22,23,24,25^

The interplay of small vessel cerebrovascular disease and neurodegeneration has particular relevance to Hispanic and non-Hispanic Black adults, who make up an increasing segment of the aging population. Inequalities across racial and ethnic groups in prevalence and incidence of AD are well documented,^26,27,28,29,30,31^ and we have hypothesized that cerebrovascular disease may be among many factors contributing to these differences. We, and others, provided evidence of increased cerebrovascular disease among community-dwelling ethnically and racially minoritized older adults^32,33,34,35^ and that its impact on cognition differs across ethnic and racial groups.^36^ In the current study, we examined whether total and regional WMH volumes were associated with a pattern of longitudinal cortical thinning that is spatially similar to what is typically seen in AD^37,38^ among a racially and ethnically diverse cohort of older adults. We hypothesized that WMH, particularly when distributed in parietal regions, would be associated with increased rates of cortical atrophy in a pattern similar to what has been observed in MCI and early progression to AD, with predominant entorhinal cortex involvement,^37,38,39^ and that these areas of increased thinning would consequently be associated with poorer memory. We further hypothesized that the association of WMH with cortical atrophy would be greater among non-Hispanic Black and Hispanic participants, reflecting either group differences in the severity of WMH and/or the influence of other health-related factors that align with race and ethnicity.^40^

## Methods

### Participants

Participants were from the Washington Heights Inwood Columbia Aging Project (WHICAP), a community-based study of cognitive aging and dementia in residents of northern Manhattan, New York, which has been ongoing since 1992. Neuroimaging substudies were introduced initially in 2004 with MRI acquired at 1.5T.^32^ Corresponding with the third WHICAP recruitment wave that began in 2010, a random subset of WHICAP participants was invited to participate in a longitudinal 3T MRI study. The current study included 303 participants from this subgroup, who had available MRI scans at 2 points, baseline and following a mean (SD) 4.1 (2.4) year interval. We compared demographic features of those included in the analysis with those of the overall WHICAP sample to examine whether there were systematic differences in representation. Participants self-identified with a race and ethnicity that included non-Hispanic White, Hispanic, non-Hispanic Black, or other. The 8 participants who identified as other were categorized as White for these analyses, although we removed them in sensitivity analyses. Participant demographic characteristics, baseline WMH volume, and cortical thickness measures are displayed in Table 1. This study was reviewed and approved by the Columbia University Medical Center institutional review board; all participants gave written informed consent. This study followed the Strengthening the Reporting of Observational Studies in Epidemiology (STROBE) reporting guideline.

**Table 1.** Descriptive Data for the Participants Included in This Study

Characteristic	Participants, No. (%) (N = 303)
Age, mean (SD), y	73.16 (5.19)
Women	181 (60)
Men	122 (40)
Education, mean (SD), y	12.80 (4.80)
Diagnostic category^a^	
NC	248 (81.85)
MCI	40 (13.20)
AD	2 (0.66)
Race and ethnicity	
White	96 (31.6)
Black	113 (37.2)
Hispanic	94 (30.9)
*APOE* ε4 allele status	
Noncarrier	202 (66.7)
Carrier	101 (33.3)
WMH volume, mean (SD), cm^3^	
Total	4.81 (5.36)
Frontal	2.04 (2.79)
Temporal	0.31 (0.14)
Parietal	1.30 (1.96)
Occipital	0.40 (0.49)
Entorhinal cortical thickness, mean (SD), mm	3.30 (0.31)

Abbreviations: AD, Alzheimer disease (clinical); MCI, mild cognitive impairment; NC, normal control; WMH, white matter hyperintensities.

^a^ Thirteen participants are missing from the diagnostic evaluation.

### Neuropsychological Testing

Participants underwent a neuropsychological examination that assessed cognitive domains of language, memory, executive function/speed of processing, and visuospatial function, as previously described.^41^ The measure of delayed recall was derived from Selective Reminding Test^42^ and was used to examine declarative memory. The diagnosis of dementia and MCI has been previously described.^43^

### MRI

Magnetic resonance images were acquired on a 3T Philips Achieva scanner at Columbia University beginning in 2011. T1-weighted (resolution, 1 mm × 1 mm × 1 mm; repetition time, 6.6 milliseconds; echo time, 3.0 milliseconds; field of view, 256 mm × 200 mm × 165 mm) and T2-weighted fluid-attenuated inversion recovery (FLAIR; resolution, 0.43 mm × 0.43 mm × 0.6 mm; repetition time, 8000 milliseconds; echo time, 332.0 milliseconds; inversion time, 2400 milliseconds; field of view, 240 mm × 240 mm × 180 mm) images were acquired in the transverse orientation.

**Figure 1.**
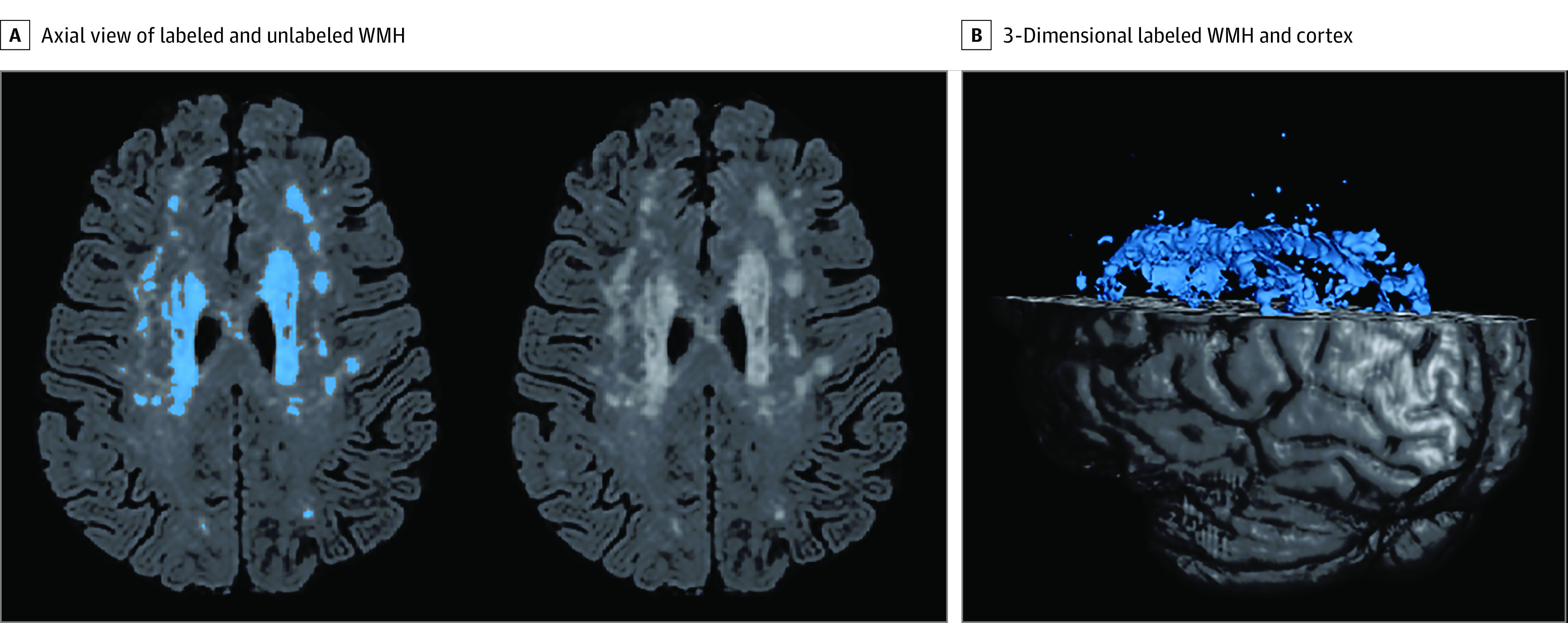
White Matter Hyperintensity (WMH) Quantification A, Axial view of a fluid-attenuated inversion recovery brain extracted image demonstrating labeled WMH with in-house developed software on the left and unlabeled WMH distribution on the right. B, A 3-dimensional rendering of labeled WMH and cortex.

Total and regional WMH volumes at time 1 were quantified with methods previously described.^6,44^ Briefly, T2-weighted FLAIR images were brain extracted, and a single Gaussian curve was fit to voxel intensity values; a threshold of 2.1 SD greater than the mean intensity value defined lower boundary of hyperintense voxels, and voxels greater than this value were labeled (Figure 1). The labeled mask was visually inspected, and false-positive labeling was manually edited. Regional WMH volumes in the frontal, temporal, parietal, and occipital lobes were derived by spatially coregistering a lobar atlas^45^ to each participant’s FLAIR image and computing the volume of labeled WMH within each brain lobe.

FreeSurfer (version 6.0) longitudinal pipeline analysis was used to quantify changes in cortical thickness between the 2 points.^46^ Briefly, an unbiased within-participant template space and image were created,^47^ and processing steps including skull-stripping, Talairach transformation, atlas registration, and surface maps and parcellations were initialized with common information from the within-participant template. The longitudinal 2-stage model was applied to each participant’s T1-weighted image at each point. Group analysis was conducted using the mri-glmfit tool within FreeSurfer, and a general linear model was fit at each surface vertex. Symmetrized percent change between baseline and follow-up scans was measured to examine the rate of change in regional cortical thickness with respect to average thickness, also defined as 100 × rate / average thickness or calculated as 100 × (thickness 2 − thickness 1) / ([time2 − time 1] × [1 / 0.5 × {thickness 1 + thickness 2}]).

### Statistical Analysis

We examined the associations of total and regional (frontal, temporal, parietal, occipital) WMH volume with regional cortical thinning, using general linear models in vertexwise analyses in FreeSurfer. We adjusted each model for age, sex/gender, *APOE* ε4 status (ε4 allele carrier), and baseline entorhinal cortical thickness (to account for baseline differences in regional thickness). In an additional analysis, we reran this model while removing baseline entorhinal cortical thickness as a covariate and report the findings in the eAppendix in the Supplement. To correct for multiple comparisons, a 2-tailed Monte Carlo simulation was applied, using a vertexwise and cluster-forming significance threshold of *P* < .01. We extracted the mean symmetrized percent change values of thickness in significant clusters that were found to be associated with WMH volumes and used these values for our secondary analyses involving memory and race and ethnicity. Symmetrized percent change is the recommended approach for 2-point longitudinal analyses of cortical thickness data within FreeSurfer^48^ and as a statistical approach for treatment comparisons.^49^ Using the statistical package SPSS version 25 (IBM Corp), we tested associations between the mean change in cortical thickness of the clusters associated with total and regional WMH volumes at baseline and delayed recall performance on the Selective Reminding Test, administered at the clinical follow-up visit that was closest to the second MRI visit. In these models, we adjusted for age, sex/gender, *APOE* ε4 status, baseline entorhinal cortical thickness, and the time between test administration and the second MRI visit. We adjusted for years of education in an additional analysis and report these findings in the eAppendix in the Supplement. We tested whether there was an interaction between race and ethnicity and baseline WMH volume on cortical thinning in clusters identified by the omnibus test. These analyses adjusted for age, sex/gender, *APOE* ε4 status, and baseline entorhinal cortical thickness. We also ran analyses stratified by race and ethnicity following the interaction models to test for associations between baseline WMH volumes and cortical thinning separately within each racial and ethnic group. In a sensitivity analysis, we excluded the participants who identified their race and ethnicity as other in testing for interactions between race and ethnicity and baseline WMH volume on cortical thinning, and we report these results in the eAppendix in the Supplement. We tested for differences in baseline WMH volumes among racial and ethnic groups by performing univariate analyses and further adjusted for age, sex/gender, and *APOE* status. We additionally tested for differences in rates of cortical thinning (symmetrized percent change) among racial and ethnic groups by univariate analyses, adjusting for age, sex/gender, and *APOE* status. For initial clusterwise inference of cortical thinning, we report only *P* values resulting from the Monte Carlo simulation procedure. For all other analyses, we report standardized and unstandardized coefficients, confidence intervals, and *P* values resulting from the regression models.

## Results

Overall, 303 participants (mean [SD] age, 73.16 [5.19] years, 181 [60%] women, 96 [32%] non-Hispanic White, 113 [37%] Non-Hispanic Black, 94 [31%] Hispanic) with longitudinal MRI scans were included in the analyses. Table 1 displays their demographic characteristics. We compared demographic features of those included in the analysis with the 5890 WHICAP participants who were not included in the analyses. Participants in the current study were younger by 4.8 years (95% CI, 4.0 to 5.6 years; *P* < .001), had more years of formal education by 3.2 years (95% CI, 2.7 to 3.8 years; *P* < .001), included a greater proportion of men (122 [40%] vs 1885 [32%], χ^2^_1_ = 8.1; *P* = .004), and had a relatively greater proportion of African American/Black participants (113 [37%] vs 1767 [30%]) and lower proportion of Hispanic/Latinx participants (94 [31%] vs 1826 [31%]; χ^2^_2_ = 25.8; *P* < .001).

### Associations Between Total and Regional WMH Volumes and Longitudinal Cortical Thinning

**Figure 2.**
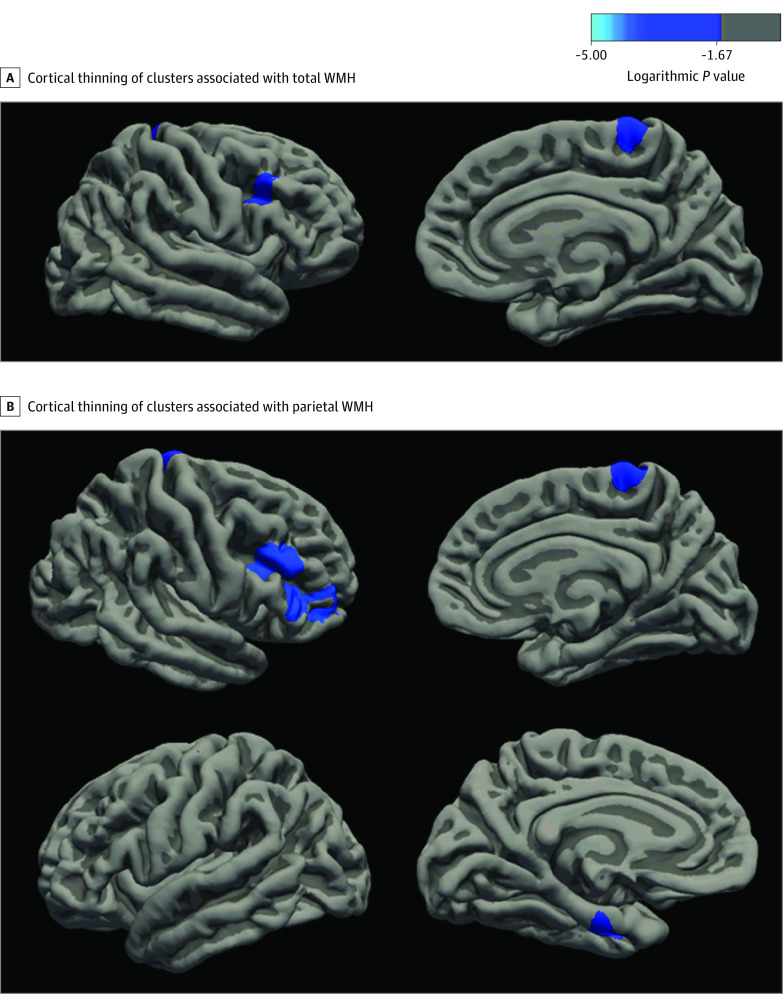
Lateral and Medial Views of the Right Hemisphere, With Cortical Thinning of Clusters Associated With White Matter Hyperintensities (WMH) A, Lateral (top left) and medial (top right) view of the right hemisphere, showing areas of cortical thinning associated with total WMH, including the caudal middle frontal and paracentral cortex. B, Lateral (top left) and medial (top right) view of the right hemisphere, showing areas of cortical thinning associated with parietal WMH, including the rostral middle frontal, pars triangularis, and paracentral cortices. Lateral (bottom left) and medial (bottom right) view of the left hemisphere, showing areas of cortical thinning associated with parietal WMH, including the entorhinal cortex. Clusters that survive a multiple comparison correction at a significance threshold of *P* < .01 are shown. The color scale indicates cluster-level logarithmic *P* values; a negative value in this legend indicates a negative correlation, whereas a positive value would indicate a positive correlation.

We tested the associations between baseline WMH volumes and longitudinal cortical thinning, adjusted for age, sex/gender, *APOE* ε4 status, and baseline entorhinal thickness. Higher total WMH volume was associated with cortical thinning in the right caudal middle frontal cortex (*P* = .001) and paracentral cortex (*P* = .04) (Figure 2A). In the total WMH model, of the covariates, individuals who carried the *APOE* ε4 allele had increased thinning of the right caudal middle frontal cortex relative to those who did not (*P* = .007). When examining regional WMH effects, higher parietal WMH were associated with cortical thinning in the left entorhinal cortex (*P* = .03), right rostral middle frontal (*P* < .001), pars triangularis (*P* < .001), and paracentral cortex (*P* = .02) (Figure 2B). In the parietal lobe WMH model, of the covariates, higher baseline entorhinal cortical thickness was associated with slower thinning of the entorhinal cortex (*P* = .03) and those who carry *APOE *ε4 had increased thinning of the right rostral middle frontal cortex relative to those who do not (*P* = .02). Frontal, temporal, and occipital WMH were not associated with subsequent cortical thinning at the multiple-comparison significance threshold of *P* < .01. These primary analyses were repeated after the removal of baseline entorhinal cortex thickness as a covariate (eAppendix in the Supplement). These results diverged only slightly from the original analyses in finding that additional thinning in the lingual cortex and temporal pole were associated with WMH.

### Associations Between Cortical Thinning and Memory

For each participant, we extracted mean values reflecting symmetrized percent change in thickness of each cluster that was associated with total and parietal WMH volumes. For the analyses in which we examined associations between cortical thinning and delayed memory recall, we adjusted for age, sex/gender, *APOE* ε4 status, the time between second MRI scan and test administration, and baseline entorhinal thickness. We found that greater cortical thinning of the right caudal middle frontal cortex (standardized β = 0.129; unstandardized *b* = 0.335; 95% CI, 0.055 to 0.616; *P* = .01) and left entorhinal cortex (β = 0.119; *b* = 0.290; 95% CI, 0.018 to 0.563; *P* = .03) were associated with worse delayed recall memory. Of the covariates, increased age at the time of the memory assessment was associated with lower delayed recall performance across models (β range, −0.204 to −0.189; *P* < .001), and increased entorhinal cortical thickness was associated with a higher delayed recall performance (β range, 0.180 to 0.208; *P* value range, .001 to .002). These analyses were repeated after further adjustment for number of years of formal education (eAppendix in the Supplement); the pattern of associations between changes in cortical thinning and memory did not change.

### Association of WMH Volumes With Cortical Thickness Across Racial and Ethnic Groups

We tested whether the association of WMH volumes and cortical thinning differed across racial and ethnic groups in those cortical thinning clusters identified as associated with WMH in the omnibus analyses. For these analyses, we adjusted for age, sex/gender, *APOE* ε4 status, and baseline entorhinal thickness. Here, we found that the association of total WMH with thinning in right caudal middle frontal cortex (β = −0.222; *b* = −0.059; 95% CI, −0.114 to −0.004; *P* = .03) and right paracentral cortex (β = −0.346; *b* = −0.155; 95% CI, −0.244 to −0.066; *P* = .001) was greater in non-Hispanic Black participants compared with White participants. The association of parietal WMH with cortical thinning of the right rostral middle frontal (β = −0.252; *b* = −0.202; 95% CI, −0.349 to −0.055; *P* = .007), right pars triangularis (β = −0.327; *b* = −0.253; 95% CI, −0.393 to −0.113; *P* < .001), and right paracentral (β = −0.263; *b* = −0.337; 95% CI, −0.567 to −0.107; *P* = .004) cortices was also stronger among non-Hispanic Black participants. The association of WMH volumes with cortical thinning was similar in Hispanic participants compared with non-Hispanic White participants. Results of these analyses stratified by race and ethnicity are reported in Table 2. Briefly, the stratified analyses were consistent with the interaction models such that the association of total WMH with thinning in the right caudal middle frontal and right paracentral cortex was most reliable in non-Hispanic Black participants. Similarly, the association between parietal WMH on thinning of the right rostral middle frontal, right pars triangularis, and right paracentral cortices was stronger in non-Hispanic Black participants compared with non-Hispanic White or Hispanic participants.

**Table 2.** Stratified Results of the Associations Between WMH and Regions of Cortical Thinning by Race and Ethnicity

Region with associated cortical thinning	Participants by race and ethnicity					
	Non-Hispanic Black		Hispanic		Non-Hispanic White	
	b (95% CI)	*P* value	b (95% CI)	*P* value	b (95% CI)	*P* value
Total WMH						
Right caudal middle frontal	−0.074 (−0.123 to −0.024)	.004	−0.027 (−0.049 to 0.005)	.09	−0.016 (−0.049 to 0.017)	.35
Right paracentral	−0.180 (−0.255 to −0.106)	<.001	−0.050 (−0.120 to 0.020)	.16	−0.027 (−0.078 to 0.024)	.29
Parietal WMH						
Right rostral middle frontal	−0.230 (−0.370 to −0.090)	.001	−0.020 (−0.108 to 0.068)	.65	−0.026 (−0.107 to 0.054)	.52
Right pars triangularis	−0.234 (−0.370 to −0.098)	.001	−0.004 (−0.100 to 0.091)	.93	0.027 (−0.034 to 0.088)	.39
Right paracentral	−0.442 (−0.640 to −0.244)	<.001	−0.108 (−0.288 to 0.072)	.24	−0.091 (−0.217 to 0.035)	.16

Abbreviation: WMH, white matter hyperintensities.

Of the covariates in these models, women had slower rate of thinning of the right caudal middle frontal cortex (β = 0.114; *b* = 0.262; 95% CI, 0.001 to 0.522; *P* = .04), right rostral middle frontal cortex (β = 0.157; *b* = 0.353; 95% CI, 0.100 to 0.605; *P* = .006), and right pars triangularis cortex (β = 0.181; *b* = 0.394; 95% CI, 0.154 to 0.635; *P* = .001) compared with men. Individuals who carry the *APOE* ε4 allele had increased thinning of the left entorhinal cortex (β = −0.171; *b* = −0.435; 95% CI, −0.717 to −0.153; *P* = .003) compared with those who do not. Higher baseline entorhinal cortical thickness was associated with increased thinning of the right paracentral cortical thickness (total WMH: β = −0.116; *b* = −0.702; 95% CI, −1.367 to −0.036; *P* = .03, parietal WMH: β = −0.128; *b* = −0.718; 95% CI, −1.342 to −0.093; *P* = .03), yet slower thinning of the left entorhinal cortical thickness over time (β = 0.137; *b* = 0.523; 95% CI, 0.098 to 0.949; *P* = .01).

### Differences in WMH and Cortical Thinning Across Racial and Ethnic Groups

We examined whether baseline WMH volumes and areas of cortical thinning associated with baseline WMH differed across racial and ethnic groups. For these analyses, we adjusted for age, sex/gender, and *APOE* ε4 status (Table 3). Non-Hispanic Black participants had numerically greater WMH volumes than Hispanic and non-Hispanic White participants, although differences were not statistically significant (Table 3). The rate of cortical thinning in areas associated with baseline WMH was greatest among Black participants (Table 3), but this was only statistically significant in the right pars triangularis cortex.

**Table 3.** Baseline Values of Total and Regional WMH Volumes and of Rates of Cortical Thinning of Clusters Associated With WMH, by Racial and Ethnic Group

Measure	Participants, mean (SD)			*F*	*P* value
	White	Hispanic	Non-Hispanic Black		
WMH, cm^3^					
Total	4.54 (5.62)	4.35 (4.88)	5.43 (5.49)	1.032	.36
Frontal	1.91 (2.70)	1.86 (2.46)	2.32 (3.10)	0.709	.49
Temporal	0.35 (0.54)	0.23 (0.32)	0.35 (0.46)	1.186	.31
Parietal	1.16 (2.14)	1.13 (1.84)	1.55 (1.90)	1.369	.26
Occipital	0.47 (0.56)	0.34 (0.38)	0.39 (0.50)	1.754	.18
SPC, total WMH					
Right caudal middle frontal	−0.12 (0.93)	−0.24 (0.75)	−0.19 (1.50)	0.943	.39
Right paracentral	−0.46 (1.44)	−0.14 (1.67)	−0.58 (2.35)	0.371	.69
SPC, parietal WMH					
Right rostral middle frontal	−0.11 (0.85)	−0.18 (0.77)	−0.25 (1.47)	1.630	.20
Right pars triangularis	−0.13 (0.63)	−0.13 (0.83)	−0.40 (1.46)	3.434	.03
Right paracentral	−0.48 (1.36)	−0.14 (1.61)	−0.59 (2.13)	0.428	.65
Left entorhinal	−0.60 (1.09)	−0.62 (1.10)	−0.49 (1.36)	0.166	.85

Abbreviations: SPC, symmetrized percent change; WMH, white matter hyperintensities.

## Discussion

We found that total WMH volume was associated with cortical thinning over 4 years in right frontal/parietal regions and that parietal WMH was associated with left entorhinal cortical and right frontal atrophy. These atrophy patterns are characteristic of neurodegeneration observed in AD, including entorhinal cortex and multiple regions in the cortical mantle that define the AD cortical signature.^37^ However, other regions demonstrating cortical thinning, including the paracentral and pars triangularis cortex, are relatively spared in AD.^50^ Furthermore, we found that among the areas identified as related to WMH volume, cortical thinning of the right caudal middle frontal and left entorhinal cortices was associated with delayed recall performance on a test of declarative memory. The association between WMH volumes and cortical thinning in several frontal and parietal regions was stronger among non-Hispanic Black participants than among White participants. The association of WMH with cortical thinning was similar in Hispanic and non-Hispanic White participants.

Our longitudinal study suggests that regional neurodegeneration is at least partially explained by the severity of small vessel cerebrovascular disease, operationalized here as WMH volume. We adjusted for baseline entorhinal cortical thickness as a marker of previous atrophy to further demonstrate that these white matter abnormalities are associated with subsequent neurodegenerative changes and are not necessarily a consequence of previous neurodegeneration.^51^

Our finding of the associations between cortical thinning of the right caudal middle frontal and the left entorhinal cortices with delayed memory has important implications for Alzheimer-like clinical symptoms. These changes in cortical thickness due to WMH may partially mediate decline in long-term memory, a typically primary, early cognitive symptom of AD.^52,53^ These results provide additional support for our previous cross-sectional study,^15^ which demonstrated that medial temporal lobe cortical thickness statistically mediates a cross-sectional association between WMH volume and memory.

The findings of increased thinning of frontal and entorhinal cortex may reflect multiple mechanisms, including the advancement of both age-related and AD-specific processes. Cortical thinning in normal aging is typically most prominent in the prefrontal cortex^54,55^ and follows an anterior-posterior gradient.^56^ We found that WMH were associated with cortical thinning in several frontal regions, demonstrating that cerebrovascular disease may be one source of age-related frontal cortical thinning. Neurodegeneration in the entorhinal cortex is among the earliest changes related to AD.^38,39^ Our previous work found a specific association of parietal lobe WMH with risk and progression of AD.^6,8,11^ Here, our finding that increased parietal WMH volume was associated with atrophy in the entorhinal cortex provides further evidence that some of the neurodegenerative changes observed in AD or attributable to AD may be the result of an AD-related pattern of cerebrovascular disease. As regions of the medial temporal cortex and parietal areas share vascular supply through the posterior cerebral artery,^57^ one possible mechanism may be that reduced vascularization of the parietal region may also consequentially affect medial temporal structures. The AD cortical signature, comprising the medial temporal, inferior temporal, temporal pole, angular, superior frontal, superior parietal, supramarginal, precuneus, and inferior frontal cortices, manifests with increased thinning in those with mild AD compared with healthy control participants.^37^ In our analyses, WMH volumes were associated with thinning of regions over time, including the rostral and caudal middle frontal cortex, overlapping with regions of the AD-cortical signature.

The lateralized findings, such as greater left entorhinal and right frontal/parietal cortical thinning, were unexpected. However, previous work found that individuals with amnestic MCI and those who progress to clinical AD show greater atrophy in the left entorhinal cortex compared with the right entorhinal cortex.^58^ Our findings suggest that some of this asymmetry may be explained by differential hemispheric impact of vascular abnormalities in the form of WMH. While the specific mechanisms remain to be examined, our study provides preliminary evidence for a vascular role in AD-related neurodegeneration.

The racially and ethnically diverse study sample allowed us to examine whether cerebrovascular disease has differential associations with neurodegeneration across groups. The associations between WMH and cortical thinning of several frontal and parietal regions were stronger in non-Hispanic Black participants compared with White participants. These observations should be taken in the context of numerically higher WMH volumes and greater rates of cortical thinning in areas associated with baseline WMH among non-Hispanic Black participants compared with non-Hispanic White participants. The findings suggest that intervention on factors that promote WMH may contribute to a reduction in race disparities in neurodegeneration and its impact on clinical outcomes.^40^ Differences in baseline WMH volume between Black and White participants were not statistically significant, which was likely due to relatively smaller samples than our previous reports that did find differences.^32,36^ Thus, the more statistically reliable interaction that revealed a greater association of WMH with cortical thinning among Black participants could also point to other health-related factors that may amplify the impact of cerebrovascular disease. For example, lifespan exposures to structural racism and its consequential impact on health may affect the association of health exposures (eg, WMH) with outcomes (eg, cortical thinning).

Future research should examine WMH in the context of AD-specific biomarkers, including β amyloid and tau, how these imaging markers interact, and the sequence of their expression leading to neurodegeneration. As WMH progressively accumulate much earlier than other AD-specific biomarkers, understanding how to prevent WMH burden by reducing modifiable risk factors can potentially lower adverse clinical outcomes associated with neurodegeneration.

### Strengths and Limitations

There are a number of strengths and limitations of the study. A notable strength is its large sample size, which allowed us to test associations between WMH and longitudinal cortical thinning, in addition to maximizing the generalizability of the findings. The incorporation of longitudinal data is another key advantage, as it provides additional support for the idea that WMH are prospectively associated with neurodegeneration in an AD-like pattern, which several cross-sectional studies^13,14,15,16,17^ on this topic suggested previously. Moreover, this study adds to our understanding of the spatial associations of WMH with regional atrophy and cognition. Additionally, the inclusion of a community-dwelling population that is multiethnic and racially diverse is a core strength to this study, as it provides greater external validity of the observed associations. In terms of limitations, although the analyses are longitudinal, we cannot establish causality of the associations definitively. Furthermore, like many observational human studies that involve neuroimaging, there may be bias with respect to which participants receive imaging at baseline and follow-up, measurement error, and insufficient longitudinal follow-up to establish long-term longitudinal outcomes.

## Conclusions

In this cohort study of community-dwelling older adults, we found that WMH, a reflection of small vessel cerebrovascular disease, were associated with subsequent cortical atrophy in regions that overlap with typical AD neurodegeneration patterns. These changes in cortical thinning were further associated with poorer delayed memory recall. The associations between WMH and cortical thinning were stronger among non-Hispanic Black older adults compared with White older adults. Cerebrovascular disease may influence risk and progression of AD by promoting neurodegeneration in an AD-specific manner, which is associated with memory decline.

## Notes

Supplement. **eAppendix. **Additional Analyses
