# Supplementary material for: Association of Regional White Matter Hyperintensities With Longitudinal Alzheimer-Like Pattern of Neurodegeneration in Older Adults
Source: JAMA Netw Open. 2021 Oct 5;4(10):e2125166. doi: 10.1001/jamanetworkopen.2021.25166 (PMC8493439; doi:10.1001/jamanetworkopen.2021.25166)
Supplement: Supplement. — eAppendix. Additional Analyses [file jamanetwopen-e2125166-s001.pdf]

Supplemental Online Content

Rizvi B, Lao PJ, Chesebro AG, et al. Association of regional white matter hyperintensities with longitudinal Alzheimer-like pattern of neurodegeneration in older adults. *JAMA Netw Open.* 2021;4(10):e2125166. doi:10.1001/jamanetworkopen.2021.25166

eAppendix. Additional Analyses

This supplemental material has been provided by the authors to give readers additional information about their work.

© 2021 Rizvi B et al. *JAMA Network Open.*

eAppendix. Additional Analyses

Eight participants identified as "Other" excluded from race/ethnicity analyses:
Eight participants from the study identified as "Other" race/ethnicity and were excluded in this sensitivity analysis. We tested for interactions between race/ethnicity and baseline WMH volume on cortical thinning in clusters identified by the omnibus test, and analyses were adjusted for age, sex/gender, APOE ε4 status, and baseline entorhinal cortical thickness. Findings remained, with the exception of one region in which non-Hispanic Black participants no longer displayed a stronger rate of cortical thinning (right caudal middle frontal), though this was also trending towards significance (β=-0.209, *b*=-0.056, 95% CI [-0.114, 0.003], p=0.06).

Primary models without adjusting for entorhinal cortex thickness as a covariate:
The primary models were rerun using the covariates of age, sex/gender, APOE ε4 status, and excluded the covariate of entorhinal cortical thickness. Total WMH was associated with increased longitudinal cortical thinning in right rostral middle frontal ($p \leq 0.001$) and paracentral cortex (p=0.04). For regional WMH, parietal WMH was associated with increased longitudinal cortical thinning in the right rostral middle frontal ($p \leq 0.001$), right pars triangularis (p=0.0002), and right paracentral (p=0.008), left entorhinal (p=0.02), and left lingual cortex (p=0.02).

Associations between cortical thinning of regions related to WMH and memory, when adjusting for education:
When adjusting for age, sex/gender, APOE ε4 status, time interval and education, the associations between cortical thinning and memory did not change. We found that greater cortical thinning of the right caudal middle frontal (β=0.125, *b*=0.325, ,95% CI [0.047, 0.603], p=0.02) and left entorhinal cortex (β= 0.121, *b*=0.295, 95% CI [0.027, 0.563], p=0.03) were related to worse delayed recall memory.

Removal of entorhinal cortical thickness as a covariate in models testing associations with cortical thinning and memory
We reran models examining the associations between changes in cortical thinning in regions found to be related to WMH and memory, and adjusting for age, sex/gender, APOE ε4 status, and the time interval, but without baseline entorhinal cortex thickness as a covariate. The findings were similar to the primary analyses: greater cortical thinning of the right caudal middle frontal (β=0.378, *b*=0.146, 95% CI [0.094, 0.663], p=0.009) and left entorhinal cortex (β=0.147, *b*=0.360, 95% CI [0.087, 0.633], p=0.01) were related to worse delayed recall memory.

Removal of entorhinal cortical thickness as a covariate in models testing differences among racial/ethnicity groups in the associations between WMH and cortical thinning
Similarly, we removed baseline entorhinal cortical thickness when testing whether the associations of WMH volumes and cortical thinning differed across racial/ethnicity groups. For these models, we covaried for age, sex/gender, and APOE ε4 status. Findings remained consistent with the primary analyses, with stronger associations between WMH volumes and cortical thinning in non-Hispanic Black participants. More specifically, we found that associations between total WMH and longitudinal thinning in right caudal middle frontal (β=-0.227, *b*=-0.060, 95% CI [-0.115, -0.005], p=0.03), right paracentral cortex (β=-0.338, *b*=-0.151, 95% CI [-0.241, -0.062], p=0.001), were greater in non-Hispanic Black participants compared with White participants. The association of parietal WMH and cortical thinning of

© 2021 Rizvi B et al. *JAMA Network Open.*

the right rostral middle frontal (β=-0.254, *b*=-0.204, 95% CI [-0.351, -0.057], p=0.007), right pars triangularis (β=-0.331, *b*=-0., 95% CI [-0.397, -0.116], p<0.001), right paracentral cortex (β=-0.258, *b*=-0.330, 95% CI [-0.562, -0.099], p=0.005) and left entorhinal cortex (β=-0.183, *b*=-0.159, 95% CI [-0.317, -0.001], p=0.04) was also stronger in non-Hispanic Black participants. The association of WMH volumes with cortical thinning was similar in Hispanic participants compared with non-Hispanic white participants.

© 2021 Rizvi B et al. *JAMA Network Open.*
